# Review of the Year Book

**Published:** 1921-04

**Authors:** 


### Review of the Year Book

THE AMERICAN YEAR BOOK OF ANESTHESIA AND ANALGESIA, 1917-1918. (Copyrighted January, 1921.) F. H. McMechan, M. D., Editor. Large quarto, bound in art buckram and printed on natural tint paper. 471 text pages, 175 illustrations. Containing a cumulative index of the pertinent literature for 1917-1918 and contributions by 84 eminent authorities. Surgery Publishing Co., Publishers, 15 East 26th St., New York City. Price, $10.

The American Year Book of Anesthesia and Analgesia (copyrighted January, 1921), covering the advances in these subjects during 1917-1918, is just at hand in its de luxe form—making as much of a typographical as a scientific appeal. Delayed in publication by the World War, it contains those methods of anesthesia and analgesia introduced to expedite military surgery, which are to find a place for themselves in civilian practice for the benefit of all concerned.

Again the editor of the Year Book has tried to make it appeal to all the progressive members of the allied professions and specialties, who, in any way, are vitally interested in current advances in the science and art of anesthesia and analgesia.

The Year Book, as a cumulative encyclopedia, provides the anesthetist, specialist, surgeon, dentist, research worker and hospital superintendent with those special advances that meet their individual requirements.

Fundamental studies in the pharmaco-physio-pathology of anesthesia and analgesia, of exceptional merit, have been included, and as many of them have a direct bearing on the clinical handling of patients submitting to operations under narcosis, they are doubly significant and valuable.

All pertinent phases of the science and practice of anesthesia and analgesia, during 1917-1918, have been covered in collated papers and researches from the most prominent international authorities and the Year Book is again a comprehensive and exhaustive post-graduate course. To those interested it is not a luxury but an every-day necessity as a reference volume.

Aside from series of contributions on complicating and safety factors of anesthesia, acidosis, blood changes, blood pressure variations, pharmaco-physio-pathological studies, both in general and local anesthesia, methods of technic, especially those developed in war surgery and the newer methods of local analgesia in surgery dentistry and the specialties, the Year Book contains a cumulative index of the pertinent literature for 1917-1918, which is invaluable to anyone making a study of any phases of these subjects and needing the necessary bibliography for reference or teaching.

